# Duloxetine-related posterior reversible encephalopathy syndrome: A case report

**DOI:** 10.1097/MD.0000000000004556

**Published:** 2016-08-19

**Authors:** Nathalie Zappella, François Perier, Fernando Pico, Catherine Palette, Alexandre Muret, Sybille Merceron, Andrei Girbovan, Fabien Marquion, Stephane Legriel

**Affiliations:** a Intensive Care Unit; b Neurology and Stroke Department; c Pharmacology Unit; d Emergency Department, Centre Hospitalier de Versailles – Site André Mignot, rue de Versailles, Le Chesnay cedex; e Paris Descartes University, Sorbonne Paris Cité–Medical School; f INSERM U970, Paris Cardiovascular Research Center, Paris, France.

**Keywords:** drug toxicity, duloxetine, hypertensive encephalopathy, MRI, posterior reversible encephalopathy syndrome, serotonin reuptake inhibitor

## Abstract

**Background:**

Posterior reversible encephalopathy syndrome (PRES) has well-established links with several drugs. Whether a link also exists with serotonin–norepinephrine reuptake inhibitor such as duloxetine is unclear.

**Methods:**

We report on a patient who developed PRES with a coma and myoclonus related to hypertensive encephalopathy a few days after starting duloxetine treatment. Magnetic resonance imaging was performed and catecholamine metabolites assayed.

**Results:**

The patient achieved a full recovery after aggressive antihypertensive therapy and intravenous anticonvulsant therapy.

**Conclusions:**

The clinical history, blood and urinary catecholamine and serotonin levels, and response to treatment strongly suggest that PRES was induced by duloxetine. Duloxetine should be added to the list of causes of PRES.

## Abstract 2

Supplemental Digital Content is available in the text

## 1 Introduction

Posterior reversible encephalopathy syndrome (PRES) is a clinicoradiological entity that has been increasingly recognized since its first description by Hinchey et al^[1]^ in 1996. We managed a patient who presented with impaired consciousness and myoclonus because of PRES complicating hypertensive encephalopathy. Duloxetine, a serotonin–norepinephrine reuptake inhibitor, had been recently added to her drug regimen. We discuss the possible causal contribution of this drug to development of hypertension and PRES.

## 2 Case presentation

**Figure 1:**
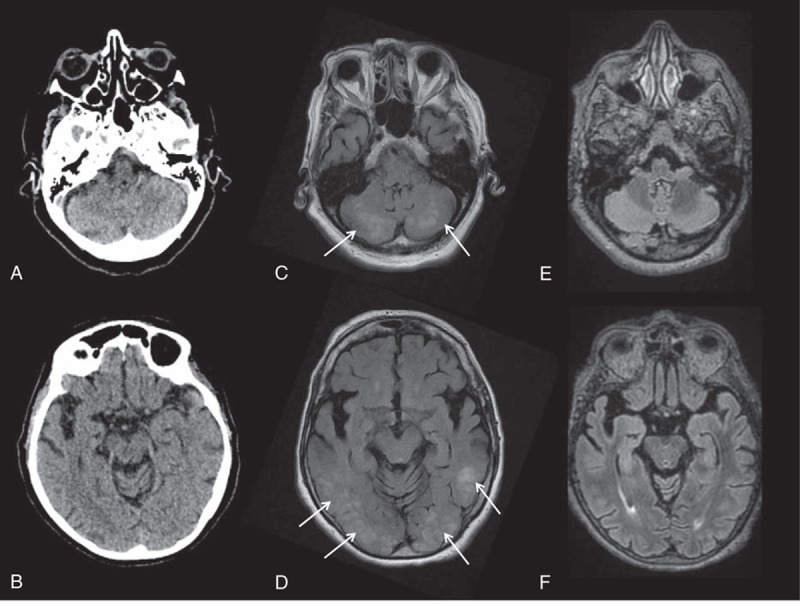
Cerebral imaging in a patient with duloxetine-related PRES. (Panels A and B) No abnormalities 5 days before the onset of PRES. (Panels C and D) FLAIR sequence showing bilateral high-signal foci in the occipital, parietal, and temporal lobes (white arrows). (Panels E and F) FLAIR follow-up sequence showing complete resolution of the abnormalities. FLAIR = fluid-attenuated inversion recovery, PRES = posterior reversible encephalopathy syndrome.

An 82-year-old woman with a history of hypertension, bipolar disorder, exertional angina, and recent spinal stenosis surgery was found at home with acute left upper limb monoplegia and right central facial palsy, following up on severe headaches. One week earlier, depressive symptoms had prompted her to visit her usual physician, who had found a normal neurological examination and a blood pressure of 188/82 mm Hg and prescribed duloxetine hydrochloride to be added to her usual regimen of celiprolol, atorvastatin, clorazepate, pregabalin, and tramadol. Two days into treatment with duloxetine, she experienced a transient impairment in consciousness. Computed tomography (CT) of the brain was unremarkable (Fig. 1A and B). Five days later she was taken to the emergency department after the acute appearance of focal motor loss. At arrival, she was conscious and breathing normally, with a blood pressure of 240/110 mm Hg, heart rate of 76 beats per minute, and blood glucose of 134 mg/dL. The limb monoplegia and facial palsy noted at her home were unchanged, and there were no other neurological abnormalities. Emergent magnetic resonance imaging (MRI) showed extensive, bilateral, high signal predominating in the temporal, parietal, occipital, and posterior fossa white matter on fluid-attenuated inversion recovery sequences (FLAIR), with corresponding low signal on T1 sequences (Fig. 1B and C). The cortical gray matter was normal. Diffusion-weighted MRI and magnetic resonance angiography (MRA) showed no vascular abnormalities.

Her neurological status deteriorated rapidly to a coma with a Glasgow Coma Scale score of 7; bilateral mydriasis; downgaze eye deviation; and diffuse abdominal, palpebral, and distal myoclonus for 30 minutes. Prompt anticonvulsant therapy consisted of intravenous clonazepam (1 mg) followed by intravenous phenobarbital (1000 mg). The myoclonus stopped but the coma remained unchanged. She was promptly intubated and mechanically ventilated. Simultaneously, the hypertensive crisis was managed with intravenous nicardipine (up to 4 mg/h). Her blood pressure stabilized at about 145/55 mmHg with oral urapidil hydrochloride 90 mg/d. Her anticonvulsant treatment was changed to levetiracetam. She recovered and was extubated on day 3 and discharged to the neurological ward on day 6.

Laboratory tests showed no metabolic disturbances. Serum duloxetine was found below lower limit of quantification after 5 days post dose. During the first 2 days in the intensive care unit, findings were normal from urinary assays of catecholamine metabolites, platelet serotonin (5-HT), and the urinary 5-HT metabolite 5-hydroxyindoleacetic acid (5-HIAA) (normetanephrine 0.75 μmoL /24 h [normal range 0.4–2.1 μmoL/24 h], metanephrine 0.34 μmoL /24 h [normal range 0.2–1.0 μmoL/24 h], 5-HT 0.10 μmoL/L [normal range 0.55–1.70 μmol/L], and 5-HIAA 18 μmoL/24 h [normal <40 μmoL/24 h]). Thus, no cause of secondary hypertension was identified.

**Figure 2:**
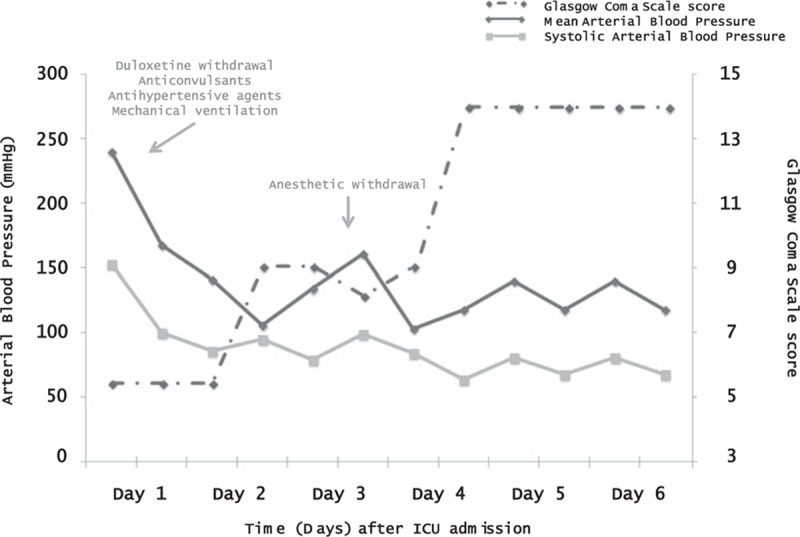
Arterial blood pressure and Glasgow Coma Scale score from the onset of PRES to ICU discharge, according to key PRES management landmarks. ICU = intensive care unit, PRES = posterior reversible encephalopathy syndrome.

Follow-up evaluations showed complete resolution of the clinical (Fig. 2) and imaging (Fig. 1 E and F) abnormalities. The final diagnosis was duloxetine-related PRES.

## 3 Discussion

PRES is a clinicoradiological entity that presents as variable combinations of seizure activity, consciousness impairment, headaches, visual impairments, nausea/vomiting, and focal neurological signs.^[1–3]^ Acute hypertension is a common, albeit not consistent, feature.^[4]^ The cerebral imaging abnormalities are often symmetric and predominate in the posterior white matter. However, the cortex may be involved also. CT is easiest to perform for first-line imaging but is often normal, although nonspecific hypodensities in a topographic distribution suggestive of PRES may be visible.^[5–7]^ MRI is superior over CT and is the key investigation for diagnosing PRES.^[7]^ The FLAIR sequence typically shows regions of high signal indicating edema. T1-weighted images demonstrate low-intensity foci in the same regions. Diffusion-weighted MRI is usually normal but the apparent diffusion coefficient is increased in the absence of ischemic complications.^[2]^ Finally, enhancement is seen in about half the cases.^[7]^ Exposure to toxic agents (Supplemental Table 1) and hypertension are the most common conditions associated with PRES. Finally, the diagnosis rests on a combination of suggestive clinical manifestations and radiological criteria occurring in an etiological setting consistent with PRES. In doubtful cases, the clinical and radiological improvement that occurs with appropriate treatment confirms the diagnosis. However, full reversibility is not consistently observed, and permanent complications or death may occur.^[8–10]^ As illustrated by our case, the severity of the clinical manifestations varies and may require ICU management and life-supporting treatments. Emergency symptomatic treatment combining anticonvulsants and drugs to control the hypertension is in order, and causative factors must be corrected without delay.^[9]^

We searched PubMed for reports of PRES associated with exposure to serotonin reuptake inhibitors, using the indexing terms “serotonin reuptake inhibitors,” “hypertensive encephalopathy,” “posterior reversible encephalopathy syndrome,” and/or “reversible posterior leukoencephalopathy syndrome.” The date limits were 1996 to March 2016 and the language limits were English, French, and Spanish. We identified only 1 case report, associated with the use of venlafaxine.^[11]^ Patient had acute hypertensive crisis, impaired consciousness, and seizures, in keeping with the clinical manifestations in our patient. He recovered fully after withdrawal of the serotonin reuptake inhibitor and symptomatic treatment to control the seizure activity and hypertension. Follow-up cerebral imaging demonstrated full resolution of the FLAIR MRI abnormalities within 6 weeks.

The two main pathogenic hypotheses are cytotoxic edema and vasogenic edema.^[12]^ Interestingly, both mechanisms may be set in motion by serotonin reuptake inhibitors.^[13]^ Excess serotonin not only exerts proinflammatory effects,^[14]^ but also directly induces contraction of the vascular smooth muscle cells and excessive capillary permeability.^[15,16]^ These actions may impair blood–brain barrier function, thereby producing the vasogenic and extracellular edema that characterizes the hypertensive encephalopathy seen in PRES.^[2]^

Several differential diagnoses may deserve discussion in patients who experience neurological events while taking serotonin reuptake inhibitors.^[17,18]^ The first is serotonin syndrome, a triad of mental changes (confusion and/or agitation), neuromuscular hyperactivity (tremor, inducible ocular and/or limb clonus, myoclonus, and/or hyperreflexia), and autonomic hyperactivity (diaphoresis and/or temperature elevation above 38°C). The diagnosis relies on the Hunter Serotonin Toxicity Criteria described by Dunkley et al^[19]^ in 2003. This syndrome may be induced when patients take simultaneously several drugs stimulating the serotonin receptors leading to drug–drug interactions. Several agents have been potentially incriminated. Among them are antimigraine agents, triptans, serotonin reuptake inhibitors, tricyclic antidepressants, monoamine oxidase inhibitors, lithium, antipsychotics, anticonvulsants, buspirone, antiparkinsonian agents, analgesics (e.g., tramadol) and even some antibiotics.^[18]^ Symptoms may start as early as 6 hours after the first drug dose.^[20]^ The treatment is nonspecific and combines immediate discontinuation of the offending drug and emergent symptomatic measures appropriate for the severity of the manifestations. The second differential diagnosis is drug discontinuation syndrome, a cause of various nonspecific neurological signs that are often benign but can occasionally mimic serious conditions such as stroke.^[21]^ The signs usually resolve after the transient administration of a different serotonin reuptake inhibitor. The third diagnosis is a serious complication of serotonin reuptake inhibitors known as reversible cerebral vasoconstriction syndrome, a clinicoradiological entity that causes severe headaches and can be revealed by seizures or focal neurological deficits.^[22]^ The diagnosis relies on the cerebral angiography finding of segmental narrowing and dilation of 1 or more cerebral arteries. A full recovery is usually achieved within 3 months after drug discontinuation and the initiation of symptomatic measures (analgesics, anticonvulsants, and antihypertensive drugs when indicated).^[23]^ Vasodilators may be helpful in the most severe cases. Complications include intracerebral bleeding and/or cerebral infarction or even PRES in 9% of cases.^[22]^ Fatal cases are exceedingly rare.^[24]^

In our patient, neither the initial clinical presentation nor the levels of platelet serotonin and catecholamine metabolites supported a diagnosis of serotonin syndrome. The plasma duloxetine level was not consistent with an overdose. MRA findings were unremarkable, and cerebral vasoconstriction syndrome was rapidly discarded.

## 4 Conclusion

The data from our patient strongly suggest a diagnosis of PRES induced by duloxetine. This agent deserves to be added to the list of causes of PRES. Early recognition of PRES and prompt management combining causative agent withdrawal and appropriate symptomatic measures are required to ensure full resolution of this potentially severe or even fatal condition.

## 5 Consent

Written informed consent was obtained from the patient for publication of this Case Report.

## Acknowledgment

The authors thank A. Wolfe for helping to prepare the manuscript.

## Supplementary Material

Supplemental Digital Content
