# Supplementary material for: Duloxetine-related posterior reversible encephalopathy syndrome: A case report
Source: Medicine (Baltimore). 2016 Aug 19;95(33):e4556. doi: 10.1097/MD.0000000000004556 (PMC5370806; doi:10.1097/MD.0000000000004556)
Supplement: Supplemental Digital Content [file medi-95-e4556-s001.doc]

**Supplemental Table 1. List of therapeutic** agents known to be associated with PRES

| Cancer chemotherapy agents (in combination)[^1-3^](#_ENREF_1) |
| --- |
| Cytotoxic agents  Alkylating agents  Cisplatin[^4^](#_ENREF_4)  Oxaliplatin[^5^](#_ENREF_5)  Carboplatin[^6^](#_ENREF_6)  Cyclophosphamide[^7^](#_ENREF_7)  Anti-metabolites  Gemcitabine[^4^](#_ENREF_4)  Cytarabine[^8^](#_ENREF_8)  Methotrexate[^9^](#_ENREF_9)  Mitotic inhibitors  Vincristine[^10^](#_ENREF_10)  Irinotecan hydrochloride[^11^](#_ENREF_11)  Etoposide[^12^](#_ENREF_12)  Vinorelbine[^13^](#_ENREF_13)  Others  L-asparaginase[^3^](#_ENREF_3)  Doxorubicin[^14^](#_ENREF_14)  Folfox[^15^](#_ENREF_15)  Vinflunine[^16^](#_ENREF_16) |
| Anti-angiogenic agents  Bevacizumab[^11^](#_ENREF_11)  Sunitinib[^17^](#_ENREF_17)  RAF kinase inhibitor BAY 43-9006[^18^](#_ENREF_18)  Sorafenib[^16^](#_ENREF_16)  Regorafenib[^19^](#_ENREF_19)  Pazopanib[^20^](#_ENREF_20)  Thalidomide[^21^](#_ENREF_21) |
| Immunomodulatory cytokines  Interferon-alpha  Interleukin-2 [^24^](#_ENREF_24) |
| Monoclonal antibodies  Rituximab (anti-CD20 )[^25^](#_ENREF_25)  Muromonab (anti-CD3)  Infliximab (anti-TNF-alfa ) [^26^](#_ENREF_26)  Pembrolizumab[^27^](#_ENREF_27)  Adalimumab[^28^](#_ENREF_28)  Anti-GD2 3F8 monoclonal antibody[^29^](#_ENREF_29)  Ipilimumab[^30^](#_ENREF_30)  Alemtuzumab[^31^](#_ENREF_31)  Cetuximab[^32^](#_ENREF_32)  Trastuzumab[^33^](#_ENREF_33) |
| Intravenous immunoglobulins[^34^](#_ENREF_34) |
| Protease inhibitors  Bortezomid[^7^](#_ENREF_7)  Carfilzomid[^35^](#_ENREF_35) |
| Anti TNF-alpha protein  Etanercept[^36^](#_ENREF_36) |
| Anti-lymphocyte globulin[^37^](#_ENREF_37) |
| Immunosuppressive agents  Anticalcineurin agents[^2^](#_ENREF_2)  Cyclosporine A  Tacrolimus (FK 506)  Sirolimus[^40^](#_ENREF_40)  High-dose corticosteroid therapy (e.g., dexamethasone and methylprednisolone)[^3^](#_ENREF_3)  Mycophenolate mofetil[^7^](#_ENREF_7)  Azathioprine[^41^](#_ENREF_41) |
| Blood transfusion[^42^](#_ENREF_42) |
| Other agents  Granulocyte-stimulating factor[^43^](#_ENREF_43)  Antiretroviral agents[^44^](#_ENREF_44)  Linezolid[^45^](#_ENREF_45)  Erythropoietin [^46^](#_ENREF_46)  Cocaine [^3^](#_ENREF_3)  Ephedra sinica (traditional Chinese remedy)[^47^](#_ENREF_47)  Intravenous contrast agents[^3^](#_ENREF_3)  Lysergic acid amide [^48^](#_ENREF_48)  Carbamazepine [^49^](#_ENREF_49)  Intravenous caffeine[^50^](#_ENREF_50)  Venlafaxine[^51^](#_ENREF_51)  Valproate[^52^](#_ENREF_52)  Ondansetron[^53^](#_ENREF_53)  Sulfasalazine[^54^](#_ENREF_54)  Mesalamine[^55^](#_ENREF_55)  Enzalutamide[^56^](#_ENREF_56)  Organophosphate[^57^](#_ENREF_57)  Desmopressin[^58^](#_ENREF_58)  Epinephrine[^59^](#_ENREF_59)  Glycerolnitrate[^60^](#_ENREF_60)  Phentermine  Licorice[^63^](#_ENREF_63)  Mephedrone[^64^](#_ENREF_64) |

**References**

**1.** Bartynski WS, Boardman JF, Zeigler ZR, Shadduck RK, Lister J. Posterior reversible encephalopathy syndrome in infection, sepsis, and shock. *AJNR Am J Neuroradiol.* Nov-Dec 2006;27(10):2179-2190.

**2.** Burnett MM, Hess CP, Roberts JP, Bass NM, Douglas VC, Josephson SA. Presentation of reversible posterior leukoencephalopathy syndrome in patients on calcineurin inhibitors. *Clin Neurol Neurosurg.* Aug 25 2010.

**3.** McKinney AM, Short J, Truwit CL, et al. Posterior reversible encephalopathy syndrome: incidence of atypical regions of involvement and imaging findings. *AJR Am J Roentgenol.* Oct 2007;189(4):904-912.

**4.** Kwon EJ, Kim SW, Kim KK, Seo HS, Kim do Y. A case of gemcitabine and cisplatin associated posterior reversible encephalopathy syndrome. *Cancer Res Treat.* Mar 2009;41(1):53-55.

**5.** Nagata Y, Omuro Y, Shimoyama T, et al. [A case of colon cancer with reversible posterior leukoencephalopathy syndrome following 5-FU and oxaliplatin (FOLFOX regime)]. *Gan To Kagaku Ryoho.* Jul 2009;36(7):1163-1166.

**6.** Vieillot S, Pouessel D, de Champfleur NM, Becht C, Culine S. Reversible posterior leukoencephalopathy syndrome after carboplatin therapy. *Ann Oncol.* Mar 2007;18(3):608-609.

**7.** Legriel S, Schraub O, Azoulay E, et al. Determinants of recovery from severe posterior reversible encephalopathy syndrome. *PloS one.* 2012;7(9):e44534.

**8.** Saito B, Nakamaki T, Nakashima H, et al. Reversible posterior leukoencephalopathy syndrome after repeat intermediate-dose cytarabine chemotherapy in a patient with acute myeloid leukemia. *Am J Hematol.* Apr 2007;82(4):304-306.

**9.** Dicuonzo F, Salvati A, Palma M, et al. Posterior reversible encephalopathy syndrome associated with methotrexate neurotoxicity: conventional magnetic resonance and diffusion-weighted imaging findings. *J Child Neurol.* Aug 2009;24(8):1013-1018.

**10.** Hualde Olascoaga J, Molins Castiella T, Souto Hernandez S, et al. [Reversible posterior leukoencephalopathy: report of two cases after vincristine treatment]. *An Pediatr (Barc).* Mar 2008;68(3):282-285.

**11.** Allen JA, Adlakha A, Bergethon PR. Reversible posterior leukoencephalopathy syndrome after bevacizumab/FOLFIRI regimen for metastatic colon cancer. *Arch Neurol.* Oct 2006;63(10):1475-1478.

**12.** Khanal P, Awan F, Nguyen V. Etoposide-induced posterior reversible encephalopathy syndrome. *Ann Hematol.* Apr 2013;92(4):561-562.

**13.** Chen YH, Huang CH. Reversible posterior leukoencephalopathy syndrome induced by vinorelbine. *Clinical breast cancer.* Jun 2012;12(3):222-225.

**14.** Kistler CA, McCall JC, Ghumman SS, Ali IA, Siddiqui AA. Posterior reversible leukoencephalopathy syndrome secondary to hepatic transarterial chemoembolization with doxorubicin drug eluting beads. *Journal of gastrointestinal oncology.* Apr 2014;5(2):E43-45.

**15.** Porcello Marrone LC, Marrone BF, Pascoal TA, et al. Posterior Reversible Encephalopathy Syndrome Associated with FOLFOX Chemotherapy. *Case reports in oncological medicine.* 2013;2013:306983.

**16.** Helissey C, Chargari C, Lahutte M, et al. First case of posterior reversible encephalopathy syndrome associated with vinflunine. *Investigational new drugs.* Oct 2012;30(5):2032-2034.

**17.** Cumurciuc R, Martinez-Almoyna L, Henry C, Husson H, de Broucker T. Posterior reversible encephalopathy syndrome during sunitinib therapy. *Rev Neurol (Paris).* Jun-Jul 2008;164(6-7):605-607.

**18.** Govindarajan R, Adusumilli J, Baxter DL, El-Khoueiry A, Harik SI. Reversible posterior leukoencephalopathy syndrome induced by RAF kinase inhibitor BAY 43-9006. *J Clin Oncol.* Oct 1 2006;24(28):e48.

**19.** Myint ZW, Sen JM, Watts NL, et al. Reversible posterior leukoencephalopathy syndrome during regorafenib treatment: a case report and literature review of reversible posterior leukoencephalopathy syndrome associated with multikinase inhibitors. *Clin Colorectal Cancer.* Jun 2014;13(2):127-130.

**20.** Chelis L, Souftas V, Amarantidis K, et al. Reversible posterior leukoencephalopathy syndrome induced by pazopanib. *BMC Cancer.* 2012;12:489.

**21.** Chow S, Cheung CS, Lee DH, Howson-Jan K, Xenocostas A. Posterior reversible encephalopathy syndrome in a patient with multiple myeloma treated with thalidomide. *Leuk Lymphoma.* May 2012;53(5):1003-1005.

**22.** Hinchey J, Chaves C, Appignani B, et al. A reversible posterior leukoencephalopathy syndrome. *N Engl J Med.* Feb 22 1996;334(8):494-500.

**23.** Kamar N, Kany M, Bories P, et al. Reversible posterior leukoencephalopathy syndrome in hepatitis C virus-positive long-term hemodialysis patients. *Am J Kidney Dis.* Apr 2001;37(4):E29.

**24.** Karp BI, Yang JC, Khorsand M, Wood R, Merigan TC. Multiple cerebral lesions complicating therapy with interleukin-2. *Neurology.* Aug 1996;47(2):417-424.

**25.** Zito JA, Lee CC, Johnson S, Singer A, Vacirca J. Reversible posterior leukoencephalopathy syndrome after rituximab. *Am J Emerg Med.* May 2010;28(4):537 e531-532.

**26.** Zamvar V, Sugarman ID, Tawfik RF, Macmullen-Price J, Puntis JW. Posterior reversible encephalopathy syndrome following infliximab infusion. *J Pediatr Gastroenterol Nutr.* Jan 2009;48(1):102-105.

**27.** LaPorte J, Solh M, Ouanounou S. Posterior reversible encephalopathy syndrome following pembrolizumab therapy for relapsed Hodgkin's lymphoma. *Journal of oncology pharmacy practice : official publication of the International Society of Oncology Pharmacy Practitioners.* Dec 11 2015.

**28.** Stetefeld HR, Lehmann HC, Fink GR, Burghaus L. Posterior reversible encephalopathy syndrome and stroke after intravenous immunoglobulin treatment in Miller-Fisher syndrome/Bickerstaff brain stem encephalitis overlap syndrome. *J Stroke Cerebrovasc Dis.* Oct 2014;23(9):e423-425.

**29.** Kushner BH, Modak S, Basu EM, Roberts SS, Kramer K, Cheung NK. Posterior reversible encephalopathy syndrome in neuroblastoma patients receiving anti-GD2 3F8 monoclonal antibody. *Cancer.* Aug 1 2013;119(15):2789-2795.

**30.** Maur M, Tomasello C, Frassoldati A, Dieci MV, Barbieri E, Conte P. Posterior reversible encephalopathy syndrome during ipilimumab therapy for malignant melanoma. *J Clin Oncol.* Feb 20 2012;30(6):e76-78.

**31.** Cooksley T, Haji-Michael P. Posterior reversible encephalopathy syndrome associated with deoxycoformycin and alemtuzumab. *The journal of the Royal College of Physicians of Edinburgh.* Sep 2011;41(3):215-217.

**32.** Palma JA, Gomez-Ibanez A, Martin B, Urrestarazu E, Gil-Bazo I, Pastor MA. Nonconvulsive status epilepticus related to posterior reversible leukoencephalopathy syndrome induced by cetuximab. *Neurologist.* Sep 2011;17(5):273-275.

**33.** Kaneda H, Okamoto I, Satoh T, Nakagawa K. Reversible posterior leukoencephalopathy syndrome and trastuzumab. *Investigational new drugs.* Aug 2012;30(4):1766-1767.

**34.** Belmouaz S, Desport E, Leroy F, et al. Posterior reversible encephalopathy induced by intravenous immunoglobulin. *Nephrol Dial Transplant.* Jan 2008;23(1):417-419.

**35.** Cai X, Bhattacharyya S, Plitt A, et al. Management of Posterior Reversible Encephalopathy Syndrome Induced by Carfilzomib in a Patient With Multiple Myeloma. *J Clin Oncol.* Jan 10 2016;34(2):e1-5.

**36.** Kastrup O, Diener HC. TNF-antagonist etanercept induced reversible posterior leukoencephalopathy syndrome. *J Neurol.* Mar 2008;255(3):452-453.

**37.** Greaves P, Oakervee H, Kon SS, Jones R, Farah N. Posterior reversible encephalopathy syndrome following anti-lymphocyte globulin treatment for severe aplastic anaemia. *Br J Haematol.* Aug 2006;134(3):251.

**38.** Schwartz RB, Bravo SM, Klufas RA, et al. Cyclosporine neurotoxicity and its relationship to hypertensive encephalopathy: CT and MR findings in 16 cases. *AJR Am J Roentgenol.* Sep 1995;165(3):627-631.

**39.** Kozak OS, Wijdicks EF, Manno EM, Miley JT, Rabinstein AA. Status epilepticus as initial manifestation of posterior reversible encephalopathy syndrome. *Neurology.* Aug 28 2007;69(9):894-897.

**40.** Bodkin CL, Eidelman BH. Sirolimus-induced posterior reversible encephalopathy. *Neurology.* Jun 5 2007;68(23):2039-2040.

**41.** Ogawa R, Oikawa T, Shijo T, Kanno S, Shibuya S, Mochizuki H. Case of posterior reversible encephalopathy syndrome due to azathioprine. *Rinsho Shinkeigaku.* Dec 23 2015;55(12):936-939.

**42.** Huang YC, Tsai PL, Yeh JH, Chen WH. Reversible posterior leukoencephalopathy syndrome caused by blood transfusion: a case report. *Acta Neurol Taiwan.* Dec 2008;17(4):258-262.

**43.** Leniger T, Kastrup O, Diener HC. Reversible posterior leukencephalopathy syndrome induced by granulocyte stimulating factor filgrastim. *J Neurol Neurosurg Psychiatry.* Aug 2000;69(2):280-281.

**44.** Ridolfo AL, Resta F, Milazzo L, et al. Reversible posterior leukoencephalopathy syndrome in 2 HIV-infected patients receiving antiretroviral therapy. *Clin Infect Dis.* Jan 15 2008;46(2):e19-22.

**45.** Nagel S, Kohrmann M, Huttner HB, Storch-Hagenlocher B, Schwab S. Linezolid-induced posterior reversible leukoencephalopathy syndrome. *Arch Neurol.* May 2007;64(5):746-748.

**46.** Delanty N, Vaughan C, Frucht S, Stubgen P. Erythropoietin-associated hypertensive posterior leukoencephalopathy. *Neurology.* Sep 1997;49(3):686-689.

**47.** Moawad FJ, Hartzell JD, Biega TJ, Lettieri CJ. Transient blindness due to posterior reversible encephalopathy syndrome following ephedra overdose. *South Med J.* May 2006;99(5):511-514.

**48.** Legriel S, Bruneel F, Spreux-Varoquaux O, et al. Lysergic acid amide-induced posterior reversible encephalopathy syndrome with status epilepticus. *Neurocrit Care.* 2008;9(2):247-252.

**49.** Furuta N, Fujita Y, Sekine A, Ikeda M, Okamoto K. [Reversible posterior leukoencephalopathy syndrome associated with carbamazepine-induced hypertension]. *Rinsho Shinkeigaku.* Apr 2009;49(4):191-193.

**50.** Ortiz GA, Bianchi NA, Tiede MP, Bhatia RG. Posterior reversible encephalopathy syndrome after intravenous caffeine for post-lumbar puncture headaches. *AJNR Am J Neuroradiol.* Mar 2009;30(3):586-587.

**51.** Edvardsson B. Venlafaxine as single therapy associated with hypertensive encephalopathy. *SpringerPlus.* 2015;4:97.

**52.** Mettananda S, Fernando AD, Ginige N. Posterior reversible encephalopathy syndrome in a survivor of valproate-induced acute liver failure: a case report. *Journal of medical case reports.* 2013;7:144.

**53.** Babi MA, Gorman MJ, Cipolla MJ, et al. Ondansetron-related hemorrhagic posterior reversible encephalopathy syndrome (PRES) following gastric bypass. *SpringerPlus.* 2016;5:18.

**54.** Ocek L, Sener U, Demirtas BS, Ozcelik MM, Oztekin O, Zorlu Y. Central-Variant Posterior Reversible Encephalopathy due to Sulfasalazine: A Case Report. *Medical principles and practice : international journal of the Kuwait University, Health Science Centre.* 2015;24(6):578-580.

**55.** Cherian A, Soumya CV, Iype T, et al. Posterior reversible encephalopathy syndrome with PLEDs-plus due to mesalamine. *Journal of neurosciences in rural practice.* Jan 2014;5(1):72-75.

**56.** Crona DJ, Whang YE. Posterior reversible encephalopathy syndrome induced by enzalutamide in a patient with castration-resistant prostate cancer. *Investigational new drugs.* Jun 2015;33(3):751-754.

**57.** Phatake R, Desai S, Lodaya M, Deshpande S, Tankasali N. Posterior reversible encephalopathy syndrome in a patient of organophosphate poisoning. *Indian journal of critical care medicine : peer-reviewed, official publication of Indian Society of Critical Care Medicine.* Apr 2014;18(4):250-252.

**58.** Wei R, Jin L, Huang J, Luo B. Desmopressin-induced posterior reversible encephalopathy syndrome. *Intern Med.* 2012;51(21):3081-3084.

**59.** Gharabawy R, Pothula VR, Rubinshteyn V, Silverberg M, Gave AA. Epinephrine-induced posterior reversible encephalopathy syndrome: a case report. *J Clin Anesth.* Sep 2011;23(6):505-507.

**60.** Kuhn AL, Huch B, Wendt G, Dooms G, Droste DW. First description of posterior reversible encephalopathy syndrome as a complication of glycerolnitrate patch following open cardiac surgery. *Acta neurologica Scandinavica.* Sep 2011;124(3):218-220.

**61.** Verro P, Wong VS. Phentermine association with a case of hemorrhagic posterior reversible encephalopathy syndrome. *Neurologist.* Apr 2015;19(4):119-120.

**62.** Wong VS, Singh H, Verro P. Posterior reversible encephalopathy syndrome in the context of phentermine use resulting in intracranial hemorrhage. *Neurologist.* Mar 2011;17(2):111-113.

**63.** van Beers EJ, Stam J, van den Bergh WM. Licorice consumption as a cause of posterior reversible encephalopathy syndrome: a case report. *Crit Care.* 2011;15(1):R64.

**64.** Omer TA, Doherty C. Posterior reversible encephalopathy syndrome (PRES) complicating the 'legal high' mephedrone. *BMJ case reports.* 2011;2011.
